# 4,4,5,5-Tetra­methyl-2-(4-pyridyl)­imidazolidin-1-oxyl-3-oxide trichloroacetic acid solvate

**DOI:** 10.1107/S1600536808016085

**Published:** 2008-06-07

**Authors:** Hong-Xian Chen, Zhong-Shu Li, Bai-Wang Sun

**Affiliations:** a Department of Chemistry, Key Laboratory of Medicinal Chemistry for Natural Resources, Ministry of Education, Yunnan University, Kunming 650091, People’s Republic of China; b Ordered Matter Science Research Center, College of Chemistry and Chemical Engineering, Southeast University, Nanjing 210096, People’s Republic of China

## Abstract

In the title compound, C_12_H_16_N_3_O_2_·C_2_HCl_3_O_2_, the imidazolidine ring adopts a twist conformation. The crystal structure is stabilized by inter­molecular O—H⋯N hydrogen bonds.

## Related literature

For related literature, see: Zhang *et al.* (2006 ▶); Ullman *et al.* (1972 ▶); Oshio *et al.* (2002 ▶); Vostrikova *et al.* (2000 ▶).
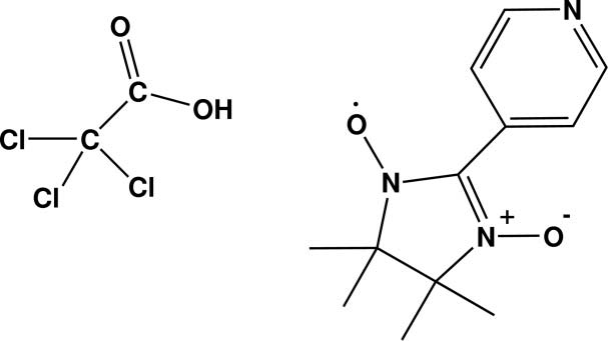


## Experimental

### 

#### Crystal data

C_12_H_16_N_3_O_2_·C_2_HCl_3_O_2_
*M* *_r_* = 397.66
Monoclinic, 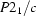

*a* = 10.003 (2) Å
*b* = 21.036 (4) Å
*c* = 9.2796 (19) Å
β = 115.33 (3)°
*V* = 1764.9 (7) Å^3^
*Z* = 4
Mo *K*α radiation
μ = 0.54 mm^−1^
*T* = 293 (2) K
0.20 × 0.20 × 0.20 mm

#### Data collection

Bruker SMART 1K CCD area-detector diffractometer
Absorption correction: multi-scan (*CrystalClear*; Rigaku, 2005 ▶) *T*_min_ = 0.895, *T*_max_ = 0.898
14888 measured reflections
3100 independent reflections
2041 reflections with *I* > 2σ(*I*)
*R*_int_ = 0.098

#### Refinement

*R*[*F* ^2^ > 2σ(*F* ^2^)] = 0.064
*wR*(*F* ^2^) = 0.132
*S* = 1.06
3100 reflections
217 parameters
H-atom parameters constrained
Δρ_max_ = 0.36 e Å^−3^
Δρ_min_ = −0.26 e Å^−3^

### 

Data collection: *CrystalClear* (Rigaku, 2005 ▶); cell refinement: *CrystalClear*; data reduction: *CrystalClear*; program(s) used to solve structure: *SHELXS97* (Sheldrick, 2008 ▶); program(s) used to refine structure: *SHELXL97* (Sheldrick, 2008 ▶); molecular graphics: *SHELXTL* (Sheldrick, 2008 ▶); software used to prepare material for publication: *SHELXTL*.

## Supplementary Material

Crystal structure: contains datablocks I, global. DOI: 10.1107/S1600536808016085/rz2217sup1.cif

Structure factors: contains datablocks I. DOI: 10.1107/S1600536808016085/rz2217Isup2.hkl

Additional supplementary materials:  crystallographic information; 3D view; checkCIF report

## Figures and Tables

**Table 1:** Hydrogen-bond geometry (Å, °)

*D*—H⋯*A*	*D*—H	H⋯*A*	*D*⋯*A*	*D*—H⋯*A*
O4—H4*B*⋯N1^i^	0.82	1.75	2.567 (7)	173

Symmetry code: (i) 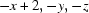.

**Fig. 1.**
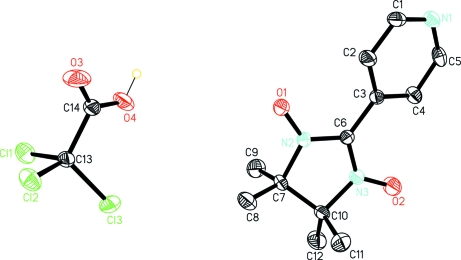
The molecular structure of the title compound with the atom-numbering scheme. All hydrogen atoms are omitted except for H4B. Displacement ellipsoids are drawn at the 30% probability level.

**Fig. 2.**
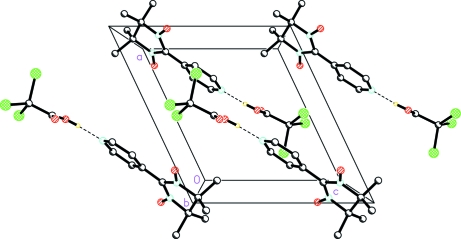
Packing diagram of the title compound viewed along the b axis. Hydrogen bonds are shown as dashed lines. All hydrogen atoms are omitted except for H4B.

## supplementary crystallographic information

### Comment

Transition metal compounds containing nitroxide radical ligands are of great interest, as these compounds play an important role in molecule-based magnetic materials (Oshio *et al.*, 2002; Vostrikova *et al.*, 2000). In order to investigate the crystal structure of such ligands, the title compound has been synthesized and its crystal structure is reported here.

In the title compound (Fig. 1), the imidazole ring adopts a twist conformation, with atoms C7 and C10 displaced by 0.218 (4) and 0.240 (4) Å respectively on opposite sides of the plane through atoms N2, N3, C6. The dihedral angle between the pyridine and the mean plane of the imidazole ring is 20.31 (27)°. This angle is smaller than that of 25.66 (15)° observed in the unsolvated compound (Zhang *et al.*, 2006). In the crystal structure, an intermolecular hydrogen bonding interaction involving the hydroxyl group of the trichloroacetic acid and the N atom of the pyridine ring is observed (Table 1).

### Experimental

4,4,5,5-Tetramethyl-2-(4-pyridyl)imidazolin-1-oxyl-3-oxide was prepared according to the published method (Ullman *et al.*, 1972). All chemicals used (reagent grade) were commercially available. 2-(4-Pyridyl)-4,4,5,5-teramethylimidazolin-1-oxyl-3-oxide (0.024 g, 0.1 mmol) was dissolved in ethanol (10 ml). Trichloroacetic acid (0.016 g, 0.1 mmol) was added slowly with stirring. The resulted solution was continuously stirred for about 30 min at room temperature and then filtered. The filtrate was slowly evaporated at room temperature over several days, to give colourless crystals suitable for X-ray analysis.

### Refinement

All H atoms were placed at calculated positions and allowed to ride on their parent atoms, with C—H = 0.93–0.96 Å, O—H = 0.82 Å, and with U_iso_(H) = 1.5 U_eq_(C, O) or 1.2 U_eq_(C) for aromatic H atoms.

### Crystal data

**Table:**

C_12_H_16_N_3_O_2_·C_2_HCl_3_O_2_	*F*_000_ = 820
*M**_r_* = 397.66	*D*_x_ = 1.497 Mg m^−^^3^
Monoclinic, *P*2_1_/*c*	Mo *K*α radiation λ = 0.71073 Å
Hall symbol: -P 2ybc	Cell parameters from 12720 reflections
*a* = 10.003 (2) Å	θ = 1.0–27.6º
*b* = 21.036 (4) Å	µ = 0.54 mm^−^^1^
*c* = 9.2796 (19) Å	*T* = 293 (2) K
β = 115.33 (3)º	Prism, colourless
*V* = 1764.9 (7) Å^3^	0.20 × 0.20 × 0.20 mm
*Z* = 4	

### Data collection

**Table:**

Bruker SMART 1K CCD area-detector diffractometer	3100 independent reflections
Radiation source: fine-focus sealed tube	2041 reflections with *I* > 2σ(*I*)
Monochromator: graphite	*R*_int_ = 0.098
Detector resolution: 8.192 pixels mm^-1^	θ_max_ = 25.0º
*T* = 293(2) K	θ_min_ = 2.5º
thin–slice ω scans	*h* = −11→11
Absorption correction: multi-scan(CrystalClear; Rigaku, 2005)	*k* = −25→25
*T*_min_ = 0.895, *T*_max_ = 0.898	*l* = −11→11
14888 measured reflections	

### Refinement

**Table:**

Refinement on *F*^2^	Secondary atom site location: difference Fourier map
Least-squares matrix: full	Hydrogen site location: inferred from neighbouring sites
*R*[*F*^2^ > 2σ(*F*^2^)] = 0.064	H-atom parameters constrained
*wR*(*F*^2^) = 0.132	*w* = 1/[σ^2^(*F*_o_^2^) + (0.0424*P*)^2^ + 1.1663*P*] where *P* = (*F*_o_^2^ + 2*F*_c_^2^)/3
*S* = 1.06	(Δ/σ)_max_ < 0.001
3100 reflections	Δρ_max_ = 0.36 e Å^−^^3^
217 parameters	Δρ_min_ = −0.26 e Å^−^^3^
Primary atom site location: structure-invariant direct methods	Extinction correction: none

### Special details

**Table:**

Geometry. All e.s.d.'s (except the e.s.d. in the dihedral angle between two l.s. planes) are estimated using the full covariance matrix. The cell e.s.d.'s are taken into account individually in the estimation of e.s.d.'s in distances, angles and torsion angles; correlations between e.s.d.'s in cell parameters are only used when they are defined by crystal symmetry. An approximate (isotropic) treatment of cell e.s.d.'s is used for estimating e.s.d.'s involving l.s. planes.
Refinement. Refinement of *F*^2^ against ALL reflections. The weighted *R*-factor *wR* and goodness of fit *S* are based on *F*^2^, conventional *R*-factors *R* are based on *F*, with *F* set to zero for negative *F*^2^. The threshold expression of *F*^2^ > σ(*F*^2^) is used only for calculating *R*-factors(gt) *etc*. and is not relevant to the choice of reflections for refinement. *R*-factors based on *F*^2^ are statistically about twice as large as those based on *F*, and *R*- factors based on ALL data will be even larger.

### Fractional atomic coordinates and isotropic or equivalent isotropic displacement parameters (Å^2^)

**Table:**

	*x*	*y*	*z*	*U*_iso_*/*U*_eq_	
Cl1	0.23783 (13)	0.11481 (6)	0.16145 (14)	0.0544 (4)	
Cl2	0.44021 (13)	0.19710 (5)	0.40583 (14)	0.0544 (4)	
Cl3	0.50692 (15)	0.06513 (6)	0.41125 (15)	0.0620 (4)	
N3	1.0715 (4)	−0.17317 (15)	0.4059 (4)	0.0378 (8)	
O1	0.9245 (3)	−0.03239 (13)	0.2203 (3)	0.0485 (8)	
N2	0.9604 (3)	−0.08379 (15)	0.2999 (4)	0.0330 (8)	
N1	1.3320 (4)	−0.10702 (18)	0.0574 (4)	0.0431 (9)	
C14	0.5057 (4)	0.1426 (2)	0.1725 (5)	0.0396 (10)	
C6	1.0677 (4)	−0.12466 (18)	0.3092 (4)	0.0326 (9)	
O4	0.5301 (4)	0.09179 (15)	0.1183 (4)	0.0594 (9)	
H4B	0.5706	0.0995	0.0598	0.089*	
C3	1.1616 (4)	−0.11841 (18)	0.2243 (4)	0.0318 (9)	
O2	1.1539 (4)	−0.22208 (15)	0.4388 (4)	0.0679 (10)	
O3	0.5285 (4)	0.19655 (16)	0.1466 (4)	0.0700 (10)	
C7	0.8677 (4)	−0.11090 (19)	0.3775 (5)	0.0365 (10)	
C10	0.9750 (4)	−0.15972 (19)	0.4916 (5)	0.0363 (10)	
C5	1.3255 (5)	−0.1617 (2)	0.1249 (5)	0.0456 (11)	
H5A	1.3779	−0.1963	0.1134	0.055*	
C13	0.4289 (4)	0.13093 (18)	0.2862 (5)	0.0369 (10)	
C4	1.2440 (4)	−0.16910 (19)	0.2113 (5)	0.0396 (10)	
H4A	1.2443	−0.2077	0.2604	0.048*	
C2	1.1702 (5)	−0.0613 (2)	0.1541 (5)	0.0513 (12)	
H2A	1.1178	−0.0260	0.1622	0.062*	
C12	0.9050 (5)	−0.2207 (2)	0.5118 (6)	0.0630 (14)	
H12A	0.8399	−0.2370	0.4090	0.094*	
H12B	0.8498	−0.2125	0.5727	0.094*	
H12C	0.9809	−0.2514	0.5667	0.094*	
C9	0.7334 (5)	−0.1403 (2)	0.2419 (5)	0.0587 (13)	
H9A	0.7651	−0.1739	0.1936	0.088*	
H9B	0.6828	−0.1084	0.1636	0.088*	
H9C	0.6677	−0.1573	0.2832	0.088*	
C11	1.0795 (5)	−0.1329 (2)	0.6540 (5)	0.0599 (13)	
H11A	1.1223	−0.0940	0.6393	0.090*	
H11B	1.1566	−0.1631	0.7088	0.090*	
H11C	1.0252	−0.1247	0.7160	0.090*	
C1	1.2570 (5)	−0.0571 (2)	0.0721 (5)	0.0537 (13)	
H1B	1.2633	−0.0186	0.0262	0.064*	
C8	0.8203 (5)	−0.0578 (2)	0.4560 (6)	0.0582 (13)	
H8A	0.9059	−0.0399	0.5413	0.087*	
H8B	0.7546	−0.0744	0.4976	0.087*	
H8C	0.7705	−0.0254	0.3786	0.087*	

### Atomic displacement parameters (Å^2^)

**Table:**

	*U*^11^	*U*^22^	*U*^33^	*U*^12^	*U*^13^	*U*^23^
Cl1	0.0439 (7)	0.0609 (8)	0.0638 (8)	−0.0087 (6)	0.0282 (6)	−0.0041 (6)
Cl2	0.0639 (8)	0.0492 (7)	0.0615 (8)	−0.0039 (6)	0.0378 (6)	−0.0122 (6)
Cl3	0.0845 (9)	0.0510 (7)	0.0616 (8)	0.0196 (7)	0.0417 (7)	0.0269 (6)
N3	0.043 (2)	0.0336 (19)	0.043 (2)	0.0057 (17)	0.0234 (17)	0.0060 (16)
O1	0.0541 (19)	0.0439 (18)	0.059 (2)	0.0196 (15)	0.0354 (16)	0.0186 (15)
N2	0.0348 (19)	0.0324 (19)	0.034 (2)	0.0051 (16)	0.0171 (16)	0.0023 (15)
N1	0.042 (2)	0.053 (2)	0.041 (2)	0.0010 (19)	0.0237 (18)	−0.0032 (18)
C14	0.032 (2)	0.052 (3)	0.037 (3)	0.001 (2)	0.016 (2)	0.007 (2)
C6	0.034 (2)	0.034 (2)	0.031 (2)	0.004 (2)	0.0153 (19)	0.0018 (18)
O4	0.071 (2)	0.069 (2)	0.061 (2)	0.0061 (19)	0.0502 (19)	−0.0005 (17)
C3	0.031 (2)	0.036 (2)	0.028 (2)	0.0002 (19)	0.0128 (18)	−0.0016 (17)
O2	0.082 (2)	0.053 (2)	0.093 (3)	0.0336 (19)	0.061 (2)	0.0324 (18)
O3	0.100 (3)	0.055 (2)	0.077 (3)	−0.011 (2)	0.058 (2)	0.0171 (18)
C7	0.030 (2)	0.044 (3)	0.042 (3)	0.001 (2)	0.022 (2)	0.000 (2)
C10	0.036 (2)	0.043 (3)	0.035 (2)	−0.001 (2)	0.021 (2)	0.0006 (19)
C5	0.037 (2)	0.046 (3)	0.061 (3)	0.003 (2)	0.027 (2)	−0.012 (2)
C13	0.041 (2)	0.033 (2)	0.043 (3)	0.0003 (19)	0.024 (2)	0.0059 (19)
C4	0.043 (3)	0.030 (2)	0.052 (3)	0.000 (2)	0.026 (2)	−0.0032 (19)
C2	0.060 (3)	0.049 (3)	0.062 (3)	0.019 (2)	0.043 (3)	0.016 (2)
C12	0.061 (3)	0.057 (3)	0.084 (4)	0.000 (3)	0.044 (3)	0.019 (3)
C9	0.036 (3)	0.079 (4)	0.051 (3)	−0.012 (3)	0.010 (2)	0.005 (3)
C11	0.055 (3)	0.075 (4)	0.045 (3)	0.007 (3)	0.017 (2)	0.002 (2)
C1	0.065 (3)	0.055 (3)	0.058 (3)	0.017 (3)	0.041 (3)	0.023 (2)
C8	0.064 (3)	0.065 (3)	0.064 (3)	0.012 (3)	0.045 (3)	0.007 (3)

### Geometric parameters (Å, °)

**Table:**

Cl1—C13	1.793 (4)	C10—C12	1.512 (6)
Cl2—C13	1.754 (4)	C10—C11	1.528 (6)
Cl3—C13	1.760 (4)	C5—C4	1.375 (5)
N3—O2	1.271 (4)	C5—H5A	0.9300
N3—C6	1.349 (5)	C4—H4A	0.9300
N3—C10	1.517 (5)	C2—C1	1.381 (5)
O1—N2	1.272 (4)	C2—H2A	0.9300
N2—C6	1.349 (5)	C12—H12A	0.9600
N2—C7	1.508 (5)	C12—H12B	0.9600
N1—C5	1.324 (5)	C12—H12C	0.9600
N1—C1	1.331 (5)	C9—H9A	0.9600
C14—O3	1.202 (5)	C9—H9B	0.9600
C14—O4	1.249 (5)	C9—H9C	0.9600
C14—C13	1.568 (5)	C11—H11A	0.9600
C6—C3	1.467 (5)	C11—H11B	0.9600
O4—H4B	0.8200	C11—H11C	0.9600
C3—C4	1.385 (5)	C1—H1B	0.9300
C3—C2	1.387 (5)	C8—H8A	0.9600
C7—C8	1.516 (5)	C8—H8B	0.9600
C7—C9	1.525 (6)	C8—H8C	0.9600
C7—C10	1.535 (6)		
			
O2—N3—C6	127.0 (3)	Cl2—C13—Cl1	108.7 (2)
O2—N3—C10	121.2 (3)	Cl3—C13—Cl1	109.1 (2)
C6—N3—C10	111.4 (3)	C5—C4—C3	119.1 (4)
O1—N2—C6	126.8 (3)	C5—C4—H4A	120.4
O1—N2—C7	121.3 (3)	C3—C4—H4A	120.4
C6—N2—C7	111.3 (3)	C1—C2—C3	119.6 (4)
C5—N1—C1	119.5 (3)	C1—C2—H2A	120.2
O3—C14—O4	129.9 (4)	C3—C2—H2A	120.2
O3—C14—C13	118.1 (4)	C10—C12—H12A	109.5
O4—C14—C13	111.9 (4)	C10—C12—H12B	109.5
N3—C6—N2	108.6 (3)	H12A—C12—H12B	109.5
N3—C6—C3	125.7 (3)	C10—C12—H12C	109.5
N2—C6—C3	125.7 (3)	H12A—C12—H12C	109.5
C14—O4—H4B	109.5	H12B—C12—H12C	109.5
C4—C3—C2	117.9 (4)	C7—C9—H9A	109.5
C4—C3—C6	121.3 (3)	C7—C9—H9B	109.5
C2—C3—C6	120.8 (3)	H9A—C9—H9B	109.5
N2—C7—C8	109.4 (3)	C7—C9—H9C	109.5
N2—C7—C9	105.3 (3)	H9A—C9—H9C	109.5
C8—C7—C9	110.4 (4)	H9B—C9—H9C	109.5
N2—C7—C10	101.0 (3)	C10—C11—H11A	109.5
C8—C7—C10	115.6 (3)	C10—C11—H11B	109.5
C9—C7—C10	114.0 (3)	H11A—C11—H11B	109.5
C12—C10—N3	109.8 (3)	C10—C11—H11C	109.5
C12—C10—C11	110.4 (4)	H11A—C11—H11C	109.5
N3—C10—C11	105.4 (3)	H11B—C11—H11C	109.5
C12—C10—C7	115.4 (3)	N1—C1—C2	121.4 (4)
N3—C10—C7	100.2 (3)	N1—C1—H1B	119.3
C11—C10—C7	114.5 (3)	C2—C1—H1B	119.3
N1—C5—C4	122.4 (4)	C7—C8—H8A	109.5
N1—C5—H5A	118.8	C7—C8—H8B	109.5
C4—C5—H5A	118.8	H8A—C8—H8B	109.5
C14—C13—Cl2	112.6 (3)	C7—C8—H8C	109.5
C14—C13—Cl3	111.1 (3)	H8A—C8—H8C	109.5
Cl2—C13—Cl3	108.5 (2)	H8B—C8—H8C	109.5
C14—C13—Cl1	106.8 (3)		
			
O2—N3—C6—N2	177.0 (4)	N2—C7—C10—C12	−143.3 (3)
C10—N3—C6—N2	−9.8 (4)	C8—C7—C10—C12	98.8 (4)
O2—N3—C6—C3	−1.2 (7)	C9—C7—C10—C12	−30.8 (5)
C10—N3—C6—C3	172.0 (3)	N2—C7—C10—N3	−25.4 (3)
O1—N2—C6—N3	179.8 (3)	C8—C7—C10—N3	−143.4 (3)
C7—N2—C6—N3	−9.0 (4)	C9—C7—C10—N3	87.0 (4)
O1—N2—C6—C3	−2.0 (6)	N2—C7—C10—C11	86.8 (4)
C7—N2—C6—C3	169.3 (4)	C8—C7—C10—C11	−31.1 (5)
N3—C6—C3—C4	13.2 (6)	C9—C7—C10—C11	−160.7 (4)
N2—C6—C3—C4	−164.8 (4)	C1—N1—C5—C4	−0.3 (6)
N3—C6—C3—C2	−166.9 (4)	O3—C14—C13—Cl2	−18.8 (5)
N2—C6—C3—C2	15.1 (6)	O4—C14—C13—Cl2	163.2 (3)
O1—N2—C7—C8	−43.0 (5)	O3—C14—C13—Cl3	−140.6 (4)
C6—N2—C7—C8	145.2 (3)	O4—C14—C13—Cl3	41.3 (4)
O1—N2—C7—C9	75.8 (4)	O3—C14—C13—Cl1	100.5 (4)
C6—N2—C7—C9	−96.0 (4)	O4—C14—C13—Cl1	−77.5 (4)
O1—N2—C7—C10	−165.3 (3)	N1—C5—C4—C3	2.2 (6)
C6—N2—C7—C10	22.9 (4)	C2—C3—C4—C5	−2.6 (6)
O2—N3—C10—C12	−41.2 (5)	C6—C3—C4—C5	177.3 (4)
C6—N3—C10—C12	145.1 (4)	C4—C3—C2—C1	1.2 (6)
O2—N3—C10—C11	77.7 (4)	C6—C3—C2—C1	−178.7 (4)
C6—N3—C10—C11	−95.9 (4)	C5—N1—C1—C2	−1.1 (7)
O2—N3—C10—C7	−163.1 (4)	C3—C2—C1—N1	0.7 (7)
C6—N3—C10—C7	23.2 (4)		

### Hydrogen-bond geometry (Å, °)

**Table:**

*D*—H···*A*	*D*—H	H···*A*	*D*···*A*	*D*—H···*A*
O4—H4B···N1^i^	0.82	1.75	2.567 (7)	173

Symmetry codes: (i) −*x*+2, −*y*, −*z*.
